# “There Are Things We Can Do and There Are Things We Cannot Do.” A Qualitative Study About Women's Perceptions on Empowerment in Relation to Fertility Intentions and Family Planning Practices in Mozambique

**DOI:** 10.3389/fgwh.2022.824650

**Published:** 2022-03-23

**Authors:** Sofia Castro Lopes, Deborah Constant, Sílvia Fraga, Nafissa Bique Osman, Jane Harries

**Affiliations:** ^1^Division of Social and Behavioural Sciences, School of Public Health and Family Medicine, University of Cape Town, Cape Town, South Africa; ^2^Epidemiology Research Unit – Institute of Public Health, University of Porto, Porto, Portugal; ^3^Department of Gynaecology and Obstetrics, Faculty of Medicine, University Eduardo Mondlane, Maputo, Mozambique

**Keywords:** women's empowerment, Mozambique, women's perceptions, fertility intentions, qualitative studies, family planning, reproductive empowerment

## Abstract

**Introduction:**

The restrictive socio-cultural norms in Mozambique limit the power of women to decide, voice, and act on their reproductive choices. This study aimed to explore women's perceptions and experiences of empowerment relating to fertility intentions and family planning practices in Mozambique, focusing on facilitators and barriers toward reproductive empowerment.

**Methods:**

Qualitative in-depth interviews were undertaken with women of reproductive age (18–49 years) in Nampula and Maputo provinces and Maputo city, Mozambique. Data collection took place between February and March 2020 in Maputo region and during August 2020 in Nampula Province. Convenience sampling was used to recruit participants from both urban and rural healthcare facilities and from within the communities serving the healthcare facilities. In Maputo city, a snowball sampling technique was used to recruit women from the community. A total of 64 women were interviewed, 39 from Maputo and 25 from Nampula. A thematic analysis was conducted with the support of NVivo12 software.

**Results:**

Several factors that hinder and facilitate women's empowerment toward fertility and family planning practices in Mozambique were identified and were interpreted within the socio-ecological model. The identified barriers included women's lack of critical consciousness and oppressive relationships. At the community and societal levels, the role of traditions, culture and gender expectations and limited access to family planning and misinformation were also important hindering factors. The facilitators of reproductive empowerment included building critical consciousness and access to economic resources at the individual level. Negative experiences at the household level were triggers of women's empowerment for family planning. Building collective power and access to information, including education, were key at the community and societal levels.

**Conclusions:**

This study identified various factors that positively or negatively influence women's empowerment journeys in Mozambique. The role of tradition, culture, and gender expectations, and oppressive relationships, were important barriers in both provinces. Women from rural areas would benefit from building of consciousness about their rights, and power to decide on their reproductive lives. Interactions with the health providers offer an opportunity to do this by favoring controlling behaviors concerning their reproductive lives, promoting social networking and levering collective power.

## Introduction

While the expansion of the family planning program in Mozambique has been effective to increase access to and the uptake of contraceptives from 17% in 2003 (1) to 35% in 2019 (2), recent evidence shows that progress toward universal access to reproductive health care is compromised when women's capacity to make their own decisions with regards to fertility intentions and family planning is constrained (3–6). In Mozambique, gender inequality is one of the main barriers to sexual and reproductive health and rights impacting women's health and lives (6, 7). The restrictive socio-cultural norms in Mozambique limit the power of women to decide, voice, and act on their reproductive choices, including negotiating with their partners or deciding when to get pregnant, how many children to have, and accessing family planning services (5, 7).

Gender equality and the empowerment of all women and girls are prioritized in goal number 5 of the Sustainable Development Goal (SDGs) (8). Women's empowerment is considered both a mechanism to tackle gender inequality and a goal in itself as empowered women more often have active participation in society, tend to acquire positive behaviors, achieve their full potential, and have better health outcomes (4, 9). In the Family Planning 2020 (FP2020—now extended to 2030) initiative, empowerment is also considered key for family planning programs and practices (10).

Family planning services with the provision of modern contraceptives are expected to be available free of charge in all public health care facilities in Mozambique. However, the supply of such commodities to health care services is often compromised by stockouts and by the limited capacity of the government to supply health services nationwide which contributes to the lack of women's access and use (6). Furthermore, there is a dearth of trained health providers on family planning who support women in informed decision-making regarding their reproductive choices.

Overall empowerment has been associated with women's increased chances of using modern contraception (11), preventing unintended pregnancy (12, 13), being able to negotiate sexual relationships (14), accessing antenatal care, and having a birth with a qualified professional (15). Despite the undeniable benefits of overall empowerment of women, the evidence from two recent systematic reviews shows inconsistent relationships between empowerment and family planning (14) and women's fertility (13). This is in part related to the diversity of definitions, conceptualization, measures, and operationalization of the term empowerment across studies (11, 16, 17).

Women's decision-making is the most studied domain of empowerment for reproductive outcomes (17). However, this is focused on decision-making about the functioning of the household rather than on reproduction, assuming that reproductive decisions take similar pathways (17), which is not supported by recent findings (18, 19). The need for tracking progress toward SDG 5.6.1: Proportion of women aged 15–49 years who make their own informed decisions regarding sexual relations, contraceptive use, and reproductive health care, has refined the use of indicators to better capture the processes of women's decision-making on their reproductive issues (20, 21). While these indicators are widely available through the Generations and Gender Survey (22) and Demographic and Health Surveys (23), allowing comparison of standardized data, they fail to capture important components of empowerment such as women's agency (24). Furthermore, empowerment is intrinsically linked to specificities of the context in which it is measured (18). Access to resources like education or employment as well as normative views, values, and women's own understanding of empowerment in different settings can shape negatively or positively the pathways of empowerment (24, 25).

Despite the recent efforts to support the conceptualization and operationalization of empowerment for sexual and reproductive health, these efforts fail to include women's own perceptions and lived experiences that look at factors that can facilitate or hinder empowerment in this sphere of a woman's life (24). This has contributed to the limited understanding of the relationship between women's empowerment and reproductive outcomes and the ability to develop effective interventions (17). The inclusion of women's views and experiences considering the specificities of the contexts where they live, could uncover important nuances of empowerment for sexual and reproductive health expanding on the conceptualization and measurements of empowerment, and support the development of relevant interventions.

This study aimed to explore women's perceptions and experiences of empowerment relating to fertility intentions and family planning practices in Mozambique focusing on facilitators and barriers women face in their journeys toward reproductive empowerment. This study builds on the existing literature on reproductive empowerment and specifically contributes to informing family planning strategies and programs in Mozambique that could enhance women's decision-making and agency on reproductive matters.

## Methods

### Study Design and Setting

Qualitative in-depth interviews were undertaken with women of reproductive age (18–49 years) in two provinces in Mozambique, Nampula in the north and Maputo in the south, and Maputo city. The selection of the study sites was based on the 2015 Demographic and Health Survey results (26) showing differences in contraceptive use and the level of empowerment of women across these provinces pointing to heterogeneity in how women perceive and experience empowerment concerning fertility intentions and family planning practices. Study participants were recruited from both urban and rural health care facilities and from within communities served by the health care facilities. The health care facilities were health centers chosen with the support of the Provincial Health Directorates (DPS—Direcção Provincial de Saúde) and the Health Directorate of the City of Maputo. In total, five health centers were selected, three urban and two rural.

The health centers are the primary level of care facilities whose services varied depending on the geographical location and level of specialized care. The selection of the health care facilities was based on the provision of the different types of contraceptive methods; the largest population served for increased diversity and, the feasibility to accessing it by the research team.

The Provincial Health Directorates liaised with the head of each health care facility for permission to conduct the study. In each health center, the research project was briefly presented by the researchers to its Director and health providers. The fieldwork team involved a lead researcher, the first author (SCL), who conducted the interviews, and a research assistant who supported translation from local languages to Portuguese.

### Study Participants

Women of reproductive age (18 to 49 years), not pregnant (confirmed verbally), attending one of the selected health facilities or living in the communities served by the health care facility were invited to participate in the study. A total of 64 women were interviewed, 39 from Maputo city and province (Health center-21; Community-18), 25 from Nampula (Health center-19; Community-6), corresponding to 41 and 23 women from urban and rural areas, respectively.

The complete list of participants and respective characteristics are presented in Supplementary Table 1. A summary of the main characteristics of the participants is presented in the Results section.

### Recruitment

The lead researcher approached women in the health center, in a group and then individually, briefly explaining the purpose of the study and what their participation would entail and, provided flyers with additional information about the study. Women interested in participating were taken to a private location in the health care facility and provided with more detailed information about the research and eligibility was confirmed. All participants signed an informed consent form after confirming their willingness to participate. The in-depth interview was conducted immediately after in the same space.

At the community level, in both Maputo and Nampula provinces, a convenience sample was used to identify and recruit eligible women with the support of the local community leaders. The community leaders invited women of reproductive age who do not attend a health facility often, who are less likely to use contraceptives, and who were available to do the in-depth interview on the scheduled days. In Maputo city, a snowball sampling technique was used since community leaders are no longer influential in the communities. In-depth interviews were conducted in the participant's home or a public space, agreed upon with the participant.

### Data Collection

Data collection took place between February and March 2020 in Maputo city and Province, and during August 2020 in Nampula Province. Due to the COVID-19 pandemic, data collection in Nampula Province had to be postponed to August 2020 when all safety measures could be put in place.

A semi-structured interview guide was developed, and the topics included were based on the published literature (17, 25, 27). Specifically, the key research questions were: how do women experience empowerment in relation to fertility intentions and family planning? What are the barriers and facilitators to the processes of empowerment for fertility intentions and family planning? In the first part of the interview, a life timeline technique was used to explore women's experiences of empowerment about their reproductive lives. The use of a timeline elicited biographical data relating to important life events, changes, decisions, and experiences (28). With the application of this technique, fertility intentions and family planning practices were mapped and explored starting at the age of women's first menstruation, going through first co-habitation, looking at moments of decision-making and processes involved around pregnancies, and power dynamics within the household and in the health services context. Other events that could indirectly have an impact such as having a paid job, death of a family member, exposure to violence or participation in a women's group or association were also explored when appropriate. The second part of the interview focused on women's perceptions around gender roles and gender power imbalances in their communities and Mozambican society. In both sections, facilitators and barriers were explored. During the interview, the questions were adjusted to the interviewee for better understanding.

Before the start of data collection, two training sessions were organized with the research assistant on the implementation of the interview guide and the informed consent process. The interview guide was piloted in the urban and rural selected locations (four interviews) in both provinces and adjustments and improvements were made in terms of language and terminology used to improve clarity.

The interviews were conducted in Portuguese, the official language of Mozambique, but, when necessary, translation to local languages was provided. The interviews had an average duration of 45 min and were audio-recorded. Interviewers kept a reflective diary.

### Data Analysis

A thematic analysis was conducted according to Braun and Clarke (29). The qualitative software package NVivo 12 was used to sort and manage data (30).

Both deductive and inductive approaches were employed in the data analysis. First, SCL coded all transcripts by identifying sentence by sentence the topics related to the theme of this work, namely barriers and facilitators of reproductive empowerment. Then a coding framework was developed together with a codebook which was validated by SF. The code framework was useful for organizing the codes that emerged inductively from the analysis under each topic. Codes and respective participant quotes with similar meanings were grouped into themes deductively based on theoretical models for reproductive empowerment (9, 24).

All interviews were transcribed verbatim and analyzed in Portuguese to prevent loss of meaning and increase the accuracy of the interpretation of the findings. At a later stage of the analysis process, when the themes and sub-themes were identified, translation of the illustrative quotes and passages was done from Portuguese into English. To guarantee the rigor and quality of data analysis, a triangulation strategy was used. The first author identified, sentence by sentence, topics related to the theme under study, and the last author collaborated in the certification of the coding framework. The barriers and facilitators' themes were then analyzed within the socio-ecological model framework (31) as a second step of the analysis. The socio-ecological model considers factors, and their interactions, at individual, relationship, community, and societal levels.

### Ethical Considerations

Ethical clearance was obtained from the Human Research Ethics Committee of the Faculty of Health Sciences, University of Cape Town (Ref: HREC 579/2019) and from the Institutional Committee of Bioethics for Health from the Faculty of Medicine/Central Hospital of Maputo, Mozambique (Ref: CIBS FM&HCM/98/2019). The DPS and the Directorate of Health of the City of Maputo approved the implementation of the study before its commencement. All participants provided written informed consent and verbal permission to the audio recording prior to the interview. Confidentiality and anonymity were ensured. Digital data and hard copies were stored in a secure place with access limited to the research team only.

## Results

Sociodemographic and economic characteristics of the participants are described in Table 1. Women from Maputo were slightly older than women from Nampula (32.5 vs. 27.8 years). They were also more educated, and a higher proportion were single. However, the mean number of years of education was similar for women in urban areas and low for women in rural areas, particularly in Nampula. Forty six percent and 12% of the participants were employed in Maputo and Nampula, respectively, of which most lived in urban areas. Contraceptive use was more prevalent among women from Maputo, with all women having used modern contraceptive methods at some point in their lives. In both provinces, women living in urban areas used more contraception than women from rural areas. In Maputo, oral contraceptives and injectables were the most prevalent methods, while in Nampula the injectable was the most preferred method. Women living in urban areas of Maputo delayed their first pregnancy on average by 3 years and had fewer children compared to women in the Maputo rural areas, however, these differences were not found between rural and urban areas in Nampula province.

**Table 1:** Sociodemographic and economic characteristics of participants from urban and rural areas of Maputo city and province and Nampula province.

	**Maputo**			**Nampula**		
	**Total**	**Urban^**a**^**	**Rural**	**Total**	**Urban**	**Rural**
	**39**	**27**	**12**	**25**	**14**	**11**
Age, mean years [±SD]	32.5 [±8.0]	33.6 [±8.1]	29.9 [±7.1]	27.8 [±9.1]	29.5 [±9.6]	26.6 [±7.7]
Education, mean years [±SD]	9.2 [±4.4]	10.6 [±4.1]	6.1 [±3.6]	8.0 [±3.5]	10.1 [±2.2]	5.2 [±3.0]
**Currently employed**						
Yes	18 (46.1)	17 (62.9)	1 (8.3)	3 (12.0)	3 (21.4)	0
**Marital status (%)**						
Single	12 (30.8)	9 (33.3)	1 (8.3)	3 (12.0)	1 (7.1)	2 (18.2)
Married or in union	23 (59.0)	14 (51.9)	9 (75.0)	20 (80.0)	11 (78.6)	9 (81.8)
Separated, divorced, or widowed	6 (15.4)	4 (14.8)	2 (16.7)	2 (8.0)	2 (14.2)	0
Age of 1st pregnancy, median [min, max]	17 [14, 27]	19.6 [15, 27]	16.5 [14, 21]	18.6 [16, 21]	18.5 [16, 20]	18.5 [17, 21]
Number of pregnancies, mean [±SD]	2.9 [±1.9]	2.6 [±1.8]	3.8 [±2.1]	4.5 [±3.6]	4.6 [±3.7]	4.3 [±3.5]
Parity, mean [±SD]	2.3 [±1.7]	1.9 [±1.5]	3.3 [±1.8]	3.8 [±2.6]	3.8 [±2.6]	3.6 [±2.6]
Number of live children, mean [±SD]	2.3 [±1.8]	1.9 [±1.6]	3.3 [±1.8]	3.2 [±2.3]	3.4 [±2.3]	2.9 [±2.1]
**Current use of contraception (%)**						
Yes	32 (82.1)	23 (82.2)	8 (66.7)	16 (64.0)	11 (78.6)	5 (45.5)
**Ever use of contraception (%)**						
Yes	39 (100.0)	27 (100.0)	12 (100.0)	14 (56.0)	9 (64.2)	5 (45.45)
**Contraceptive method currently used (%)**						
Oral contraceptive	9 (23.1)	8 (29.6)	1 (8.3)	2 (8.0)	1 (7.1)	1 (9.1)
Injection	9 (23.1)	6 (22.2)	3 (25.0)	13 (52.0)	9 (64.3)	4 (36.4)
Intrauterine device	2 (5.1)	2 (7.4)	0	0	0	0
Implant	3 (7.7)	2 (7.4)	1 (8.3)	1 (4.0)	1 (7.1)	0
Condom	8 (20.5)	5 (18.5)	3 (25.0)	0	0	0
No use	7 (17.9)	4 (14.8)	3 (25.0)	9 (36.0)	3 (21.4)	6 (54.5)

*^a^Includes participants from Maputo city and urban area of Maputo province*.

Several factors that hinder and facilitate women's empowerment toward fertility and family planning practices in Mozambique were identified by exploring women's perceptions and lived experiences. These factors were organized in themes under Barriers and Facilitator's topics separately. Empowerment is a multilevel and dynamic process of gaining power (32), that involves the individual and its interactions within the household to the societal structures. Given these characteristics, the results of this work were interpreted within the socio-ecological model (31). Table 2 summarizes the themes found under barriers and facilitators and respective levels from the socio-ecological model. A detailed description of the themes is provided below, illustrated with participants' quotes.

**Table 2:** Barriers and facilitators themes for women's empowerment regarding fertility intentions and family planning.

**Themes**	**Socio-ecological model levels**
**Barriers**	
**Lack of critical consciousness**	
- No existence of choice - Support of current system - Negative perception of gender power changes - Limited aspirations	Individual
**Oppressive relationships**	
-Controlling behavior of husband -Controlling behavior of family members -Fear of consequences	Relationship
**Role of traditions, culture, and gender expectations**	
- Early marriage and motherhood - Gender roles/activities division in the household	Societal
**Limited access to family planning and misinformation**	
- Lack or delay in receiving information - Myths and misinformation	Societal
**Facilitators**	
**Access to economic resources**	
- Employment (paid) - Access to loans and credit - “Xitique”^a^	Individual
**Building critical consciousness**	
- Opening the mind - Change in self-perception (self-value) - Aspirations - Voicing their choices	Individual
**Negative experiences as triggers of empowerment**	
- Adverse events that lead women to take control	Relationship
**Building collective power**	
- Social networks of support (e.g., associations, women's groups) - Role models	Societal
**Access to information**	
- Access to education - Access to family planning services and information	Societal

*^a^Xitique is an informal saving and credit mechanism at the community level, organized among several people who know each other*.

Although similar results were found across Maputo and Nampula regions, some nuances were captured. Overall, the barriers women faced or perceived seemed to be influenced by the setting women live in (urban or rural). Women's education and marital status seemed to shape what participants considered as a facilitator. These differences were highlighted in the results below where appropriate.

### Barriers

#### Lack of Critical Consciousness

Women's critical consciousness can be described as the process of questioning how power inequalities operate in their lives and having a sense of self-value and entitlement (9, 32). Regionally, most women from Nampula and some from Maputo, more often living in rural areas, did not question if their decision-making ability regarding their reproductive lives was being shaped by power imbalances or oppressive systems. Overall, these women were supportive of the current social organization and tended to live within the social expectations where marriage, motherhood, and gender roles were highly valued, and where the freedom and power gained by women were negatively perceived. The perception of choices in life was also limited or non-existent among these women.

This young woman from Nampula illustrates the prevailing perceived benefit of being married and the perceived value of motherhood:

“It is not good for a woman to live alone. It is important to have a husband. (…) I have never done family planning and I don't want to. I want to get pregnant again, but I haven't been able to. If God allows, I will have at least 4 children.” (Nampula, rural area, 21, single, 1 child).

Similarly, the following women show discontent toward the possible choice of other women to decide or take control over their own lives:

“Women nowadays, they want to do things first for themselves and then they think if they get married, they can go back to their houses. They don't need to stay (married). Being married is not the same as it was before. I don't know what is happening.” (Maputo, rural area, 43, 4 children).

“We women, don't respect men nowadays. If I don't respect my husband, he will also not respect me. If I do the things he doesn't want me to do, I am not respecting him.” (Nampula, rural, 20, single, 1 child).

#### Oppressive Relationships

In their relationship with male partners or other family members, women often reported oppressive behaviors which were key in how women framed their fertility intentions and use of family planning as well as framed other areas of their lives. While in Nampula, urbanized and educated women reported more often the hindering effect of oppressive relationships, that was not observed in participants from Maputo. A woman from Maputo city described her husband's behavior in the face of an unwanted pregnancy.

“When he found out I was pregnant, he (the husband) was very angry with me. I remember him saying “I spoke to my godmother, and she says you should terminate the pregnancy.” But he was lying. He would say: ”We just got married and you are already pregnant, how can you be pregnant¿‘ Those were very difficult times for me. He had no time for me and he started seeing other people from our neighborhood. That was so difficult. I was very sad.” (Maputo city, 42, married, 1 child).

Out of fear of consequences including threats of abandonment, loss of social value, verbal or physical violence or name shaming, women did not feel capable of challenging such oppressive environments or behaviors. Experiences of threat and abandonment were described by these participants.

“At home, my husband makes the decisions. I am afraid of making decisions although he has never bitten me.” (Nampula, rural area, 22, married, 3 children).

“For example, in this question about having children. If a woman doesn't want to have children now, what should she do? She goes to family planning and chooses a contraception method. And the husband only finds out later. The first consequence is that the husband no longer sees the woman as obedient. He would say: “You made the decision of not having children by yourself and you did not ask me if I want children or not. I am going to have children with someone else.” Because she decided not to have children for 5 years without his authorization, he is punishing her.” (Maputo city, 34, married, 2 children).

#### Role of Tradition, Culture, and Gender Expectations

Traditions and cultural rituals strongly embedded early marriage and childbearing in girls and young women, further promoted by the cultural belief that this is the natural and only pathway for them. The influence of traditions and rituals is explored concerning women's sexual and reproductive choices.

“From my experience within my ethnicity, if a girl reaches maturity, that is when she has her first menstruation, she is considered a grown-up and capable of having sexual intercourse with someone. She feels that she can have these sexual encounters because she has grown. It is because of the teaching in the initiation rituals that she feels she can have sexual intercourse with any person, it does not matter the age. (Maputo city, 34, married, 2 children).

In their roles as wives and mothers, women were often limited to household chores and disempowered from reproductive choices while men's power positions were prioritized. Male power positions were related role as provider and income generator:

“The man is the one who provides to you while you stay home. He gives you money to buy diapers… So, if he tells you “I want a child,” he is the one who knows because a child is his responsibility. For example, if the child is ill, he must give you money for the hospital, for the diapers, for the food, for the groceries. If he wants a child, you must give him, there is no other way.” (Maputo city, 24, single, 1 child).

However, for some women, even when the man was given the decision-maker role, it was acceptable to conceal family planning practices from their husbands. As a woman from Maputo described, different rules can be applied to family planning practices.

“A woman must obey her husband but that (family planning) is different. There are things we can do and things we cannot do” (Maputo, rural area, 25, married, 3 children).

Families' control over women's lives was also enabled by traditions and norms in both provinces. In Nampula, parents were involved in arranging a marriage or approving marriage for their daughters, as this woman recalls.

“I had a boyfriend of my age but then I got married to a friend of my uncle. He came to the house to ask my parents (for marrying her) and they accepted it.” (Nampula, rural area, 22, married, 3 children).

#### Limited Access to Family Planning and Misinformation

In Maputo, most women heard about or were offered modern contraceptives after having their first child. In Nampula, women's reported experiences seem to indicate a longer delay. A study participant explained how only after three pregnancies she obtained information about family planning.

“With my first three children I didn't use any contraceptives but after that, I got information about family planning at the hospital and some other projects in the community, so I decided to start doing contraception. I decided and just told my husband.” (Nampula, rural area, 44, married, 7 children).

Women living in urban areas of Nampula and Maputo reported other factors that also played an important role in discouraging women from practicing family planning, which were misinformation and myths about modern contraception. A woman described what she has heard men saying about contraceptives including perceived infertility due to injectable contraceptives.

“Men say that when a woman gets an injection (contraceptive) she will “burn” her tummy, “burn” future children as she won't be able to get pregnant again. So, they don't want women to “burn” their tummies, they want women to have children, all the children that God has given to them. All.” (Nampula, urban area, 32, married, 4 children).

### Facilitators

#### Access to Economic Resources

For most women paid employment was one of the most important steps toward achieving financial independence. Employment was associated with increased power for negotiation and having a voice about childbearing and family planning.

“To have a job as well as to farm the land can help a woman. If a woman works, she can make decisions. If a woman brings money, she can make decisions.” (Nampula, rural area, 23, married, 2 children).

“From my experience most women these days do not want to have children early due to the socioeconomic contexts they live in. The living conditions. Nowadays we value living conditions. If I have a child today and I am not working, what will I be able to provide to my child?” (Maputo city, 34, divorced, no children).

A resourceful and widely used practice was the “xitique,” an informal saving and credit mechanism at the community level, organized among several people (usually women) who know each other. Although this practice was very important for women's financial autonomy and building their small businesses, this was not enough to ensure financial independence. The participants from Nampula highlighted the need for institutional or government programs and funding that could support women in the development of their own businesses, give access to and create job opportunities, and facilitate access to bank loans.

“In my neighborhood, most women are street vendors, but they cannot expand their businesses because they don't have access to credit from the banks. They don't have a way to get it. They live with the “xitique,” so I would like to have that opportunity for women (to have access to bank loans).” (Nampula, urban area, 23, divorced, 1 child).

#### Building Critical Consciousness

In both regions, women mentioned the need for women to open their minds or gain consciousness of their potential, their capabilities, and self-value. This was related to changing their self-perception, questioning the status quo that perpetuates gender roles and norms, and building confidence to communicate and voice their choices openly within their relationships and in the community. These views, presented below, were often shared among single and more educated women.

“A woman must have self-esteem; she needs to feel that she is capable of doing the things she wants. Things that men do. She needs opportunities and then she will end up liking herself more. Once a woman gets a little something, she starts feeling stronger.” (Nampula, urban area, 20, single, no children).

“It would be great if we could speak, if we could speak with our husbands, with the people from the neighborhood... If we could have a voice.” (Maputo, rural area, 25, married, 3 children).

#### Negative Experiences as Triggers of Empowerment

Within the household, in the relationship with their partners and family members, adverse events seem to be a triggering factor for the empowerment of women in relation to their fertility and family planning. In the face of such negative or adverse situations, like abusive or controlling behaviors, it was acceptable for a woman to take control of her life, particularly with reproductive matters, as explained by these participants.

“There are many men who are aggressive. What they want, you must give them (sexually). So having family planning (available) helps us a lot.” (Maputo, rural area, 21, married, 2 children).

“A woman can make decisions if there are conflicts or problems at home, when there is violence, when there is no understanding between the woman and the husband.... so a woman should leave that and take care of her own life. I agree that a woman must make their own decisions when it is to avoid the worsening of the situation (between her and her husband).”(Nampula, urban area, 36, married, 5 children).

#### Building Collective Power

Only women from Maputo region, mostly single, identified social support and role models from peers as a key resource in the empowerment process. This included safe spaces, like women's associations or groups where women can come together to share experiences, find support, and debate. When engaging with each other, women can have an influencing role among themselves. It is not only those in power positions, such as the ones in the government, that serve as role models but also women from the community whose different pathways become inspirational and an example to follow by others at a local level. A study participant described her experience attending sessions at a women's association in Maputo.

“I attend a group once a week, where women get together to discuss women's issues. From maternity to society, all sorts of things. And for me, it is important to know that I am not alone, that there are other women that also don't comply with the social expectations, that are living their lives the way they want to. But within that association, there are many women who think very differently from me, and they are there and there is debate. So, I think it is important for a person, for a woman to listen to both, different sides and make their choices in life.” (Maputo city, 29, single, no children).

#### Access to Information

Access to information was especially highlighted by women living in urban areas in both regions. Education was amongst the most emphasized aspects mentioned by women as a prerequisite required for their progression toward empowerment concerning fertility and family planning practices. Creating conditions for girls and young women to access schools and ensuring these are close to their communities and safe for them to attend was linked to preventing early marriage and motherhood, and to future opportunities, as this participant from Nampula highlighted.

“We need schools in the neighborhood so women's lives can improve. I believe it is mostly schools that we need. Advise our daughters to go to school and to not get married early” (Nampula, urban area, 30, married, 6 children).

For women in Maputo, schools are the place where thinking processes and thought are stimulated, and this was key for women's empowerment in the future.

“I think school. The more schooling, the more education, the more the capacity we have to make ourselves be heard and stand for something we want. I cannot defend something that I don't know or can't understand. I need the knowledge to be able to do it... I need information.” (Maputo, urban area, 33, married, 3 children).

Access to family planning services was also considered crucial for women's empowerment for fertility and family planning in both regions. At the health facilities and in the interactions with health providers women learned about the advantages and disadvantages of the different contraceptive methods, which they found useful for their decision-making regarding family planning practices. A woman from Nampula explained how counseling from health providers provided her with confidence in choosing a contraceptive method.

“The health providers first explain the advantages and disadvantages of family planning. They give you confidence... so I trust that health provider and choose a contraceptive method.” (Nampula, urban area, 20, single, no children).

Notwithstanding, women highlighted the need to make information about family planning and contraceptive methods more available in various places, namely talks at the health center and sensitization campaigns at the community level. Furthermore, some women from Maputo mentioned the importance of personalized counseling, in a private environment, as stated by this participant.

“Yes (they give us information) but not the way it should be. When you are alone, you can feel comfortable talking if you have a problem. Usually, there are other people (patients) there (consultation room) at the same time and that does not make it easy.” (Maputo, urban area, 20, married, 1 child).

## Discussion

This study explored women's perceptions and experiences of empowerment in relation to their fertility intentions and family planning practices, identifying important barriers and facilitators for the empowerment process in Mozambique. Overall, these findings reinforce the importance of the multilevel nature of empowerment by showing how the different barriers and facilitators operate at different levels of a woman's life. Understanding such dynamics can support the development of comprehensive and more effective interventions for women's reproductive empowerment.

The findings of this work represent the perspectives of women from different sociodemographic, and geographical backgrounds, and most importantly it reports on the views of single women, largely absent in the empowerment literature (11, 14, 24). Women who took part in the study were at different points in their empowerment journeys, therefore their experiences and perceptions of barriers and facilitators were not always shared among them. The link between levels of empowerment and how women perceive and experience barriers and facilitators to empowerment deserve further analysis in future research so mechanisms of empowerment can be better understood. Furthermore, it would be interesting to compare our results with those from other studies in the African context about women's perceptions of barriers and facilitators of reproductive empowerment, however, to our knowledge, there is a lack of studies on this topic. Notwithstanding, our study could support the interpretation of quantitative indicators widely used to measure reproductive empowerment such as those included in the monitoring of the SDG 5.6.1, by providing contextual aspects and identifying structural issues (20).

At the regional level, this study captured important differences in how women perceive and experience barriers and facilitators to empowerment in relation to reproduction (25). Women from the Nampula region were, in general, more submissive to male power and gender expectations, where childbearing and marriage are highly valued, and men are responsible for fertility decisions. Women in Nampula were less willing to use modern contraceptives, also observed in other studies (6, 26). Distinct and prevailing social and gender norms, as well as strong embedded traditions in Nampula region, could in part explain the differences between regions. In addition, the limited access to information and health services with a good supply of contraceptives in the region could also play an important role in the low uptake of contraception (33). Our findings also seem to suggest that women's perception and experience of the barriers and facilitators to empowerment are shaped by other factors such as living in a rural or an urban setting, women's educational level, and marital status. This is aligned with the literature about social determinants of women's reproductive empowerment (18, 20).

Similarly to what has been described in other studies, the barriers to women's empowerment regarding fertility and family planning, are embedded in traditions, culture and gender roles as well as accepted oppressive behaviors toward women and limited access to services and information (24, 32, 33). Contrary to the findings from a study involving other African countries (24), this study showed that family members were associated with greater pressure for women's marriage and childbearing in Mozambique, often not supporting women's family planning practices (34). These barriers work toward keeping control over women's reproduction and at the same time, imposing social expectations related to women's role associated with their childbearing and marriage capacity. Often the fear of consequences from challenging these norms prevents women from embarking on empowerment journeys, contributing to the exposure to harmful situations or the use of contraceptives covertly (24, 34, 35).

An important barrier identified in this study was the lack of critical consciousness of women. The results showed that in some cases women themselves perpetuate oppressive and gender bias traditions as this is also where women can find their voice and exercise some form of power over other women (6). The development of critical consciousness is an essential resource or precondition to trigger empowerment processes (9, 11, 36), also identified in this study.

Beyond education, access to information, financial independence, empowerment resources amply described in the literature, this study identified other facilitators that deserve further attention in research and interventions. Taking part in social networks allows women to come together and share experiences which contributes to breaking oppressive beliefs and expectations among women themselves and simultaneously promoting collective power (32, 36). For example, evidence from Mozambique suggests that living in contexts where open discussion and conversations about family planning take place has a positive influence on the use of contraceptives among women (37).

Conversely, being exposed to negative events, either abusive, controlling behaviors or general neglect of the household by their partners, was also identified as a trigger for women taking control over their reproductive lives (18). Although this could be connected to other facilitators of empowerment such as being conscious of one's rights, having a sense of injustice, or having different options in life (38), further research should be conducted to understand how these negative events influence women's empowerment in relation to their fertilities and family planning practices.

Health providers were one of the main sources of information regarding family planning and modern contraception methods for women in Mozambique. Women showed general satisfaction with the family planning services and the interactions with the health providers. More private and personalized family planning counseling, as well as more information, were perceived as a need by women, which could represent an opportunity to include topics namely women's right to bodily autonomy, choosing and decision-making processes, and having other options in life that are beyond childbearing which can contribute to reproductive empowerment (3).

### Strengths and Limitations

To our knowledge, this study is the first to explore women's perceptions and lived experiences of empowerment in relation to fertility and family planning practices in Mozambique. It also places women at the center of empowerment, including single women, often overlooked, giving them a voice and expands on recent conceptual frameworks of empowerment for sexual and reproductive health. Notwithstanding, this study has some limitations. First, convenience sampling was used to recruit participants, both at the health facility and at the community level and this could have introduced bias in the selection of participants. Women who attended the health center or were known to traditional leaders could be more familiar with contraception methods and more aware of their choices than those not captured by the recruitment process. However, the in-depth interviews captured both personal experiences and more broad views of women and their experiences in Mozambican society, which could have minimized the potential selection bias. Secondly, this study failed to include male perspectives who are often the gatekeepers of decisions related to women's reproduction in Mozambique, and the views of community stakeholders whose influence on social and gender norms is still relevant. Moreover, it did not explore in-depth men's roles and responsibilities in reproductive matters. However, whilst men's roles and responsibilities were not included in the paper, this work explored empowerment for fertility intentions and family planning practices in relation to gender power dynamics. Notwithstanding, this should be further analyzed in future research.

## Conclusions

This study identified various factors at individual, societal and structural levels that positively or negatively influence women's empowerment journeys in Mozambique. These factors seemed to be influenced by women's region and place of living, urban vs. rural, as well as women's education and marital status. The role of tradition, culture, and gender expectations, as well as oppressive behaviors from partners or other family members, were important barriers for women from both provinces. Women from rural areas, particularly from Nampula, would benefit from building of consciousness about their rights, capacity, and power to decide on their reproductive lives. Interactions with the health providers offer an opportunity to do this by favoring controlling behaviors concerning their reproductive lives, promoting social networking and levering collective power and action.

## Data Availability Statement

The original contributions presented in the study are included in the article/Supplementary Materials, further inquiries can be directed to the corresponding author.

## Ethics Statement

The studies involving human participants were reviewed and approved by Human Research Ethics Committee of the Faculty of Health Sciences, University of Cape Town; Institutional Committee of Bioethics for Health from the Faculty of Medicine/Central Hospital of Maputo, Mozambique. The patients/participants provided their written informed consent to participate in this study.

## Author Contributions

SCL, DC, SF, and JH conceptualize the study. SCL designed the study, analyzed the data, and prepared the initial draft of the paper. SF supported data analysis and provided inputs. JH, SF, DC, and NO reviewed and substantially edited the manuscript. All authors read and approved the final manuscript.

## Funding

We gratefully acknowledge the PhD grant from Fundação para a Ciência e Tecnologia to SCL (SFRH/BD/146625/2019) and the contract to SF (CEECIND/01516/2017). We also thank the Faculty Research Committee (FRC) of the Faculty of Health Sciences of the University of Cape Town for the Post Graduate Research Training Grant (FRC Award 2019) to SCL.

## Conflict of Interest

The authors declare that the research was conducted in the absence of any commercial or financial relationships that could be construed as a potential conflict of interest.

## Publisher's Note

All claims expressed in this article are solely those of the authors and do not necessarily represent those of their affiliated organizations, or those of the publisher, the editors and the reviewers. Any product that may be evaluated in this article, or claim that may be made by its manufacturer, is not guaranteed or endorsed by the publisher.
