# Supplementary material for: “There Are Things We Can Do and There Are Things We Cannot Do.” A Qualitative Study About Women's Perceptions on Empowerment in Relation to Fertility Intentions and Family Planning Practices in Mozambique
Source: Front Glob Womens Health. 2022 Mar 23;3:824650. doi: 10.3389/fgwh.2022.824650 (PMC8983837; doi:10.3389/fgwh.2022.824650)
Supplement: Supplementary file 1 [file Table_1.DOCX]

**Supplementary material**

Table S1: Sociodemographic characteristics of the participants from urban and rural areas of Maputo (including Maputo city) and Nampula provinces.

| **#** | **Region** | **Local** | **Site** | **Age**  **(years)** | **Civil status** | **Education (years)** | **Employed** | **Age of 1st pregnancy** | **Number of pregnancies** | **Type of contraception currently used** | **Ever use of contraception** | **Type of contraception previously used** |
| --- | --- | --- | --- | --- | --- | --- | --- | --- | --- | --- | --- | --- |
| 1 | M | Urban | HC | 32 | Married | 15 | yes | 26 | 2 | Pill | yes | Pill |
| 2 | M | Urban | HC | 34 | Married | 16 | no | 24 | 3 | Pill | yes | Pill and condom |
| 3 | M | Urban | HC | 24 | single | 10 | yes | 20 | 1 | Pill | yes | Implant |
| 4 | M | Urban | HC | 25 | Married | 7 | no | 17 | 2 | Injection | yes | Injection |
| 5 | M | Urban | HC | 34 | Married | 17 | yes | 27 | 2 | Pill | yes | Pill |
| 6 | M | Urban | HC | 25 | Single | 16 | yes | na | 0 | Pill and condom | yes | Condom |
| 7 | M | Urban | HC | 42 | Married | 9 | no | 23 | 1 | Injection | yes | Pill |
| 8 | M | Urban | C | 29 | Single | 16 | yes | na | 0 | Condom | yes | Pill |
| 9 | M | Urban | C | 37 | Single | 12 | yes | * | 1 | Condom | yes | Pill |
| 10 | M | Urban | C | 25 | Single | 12 | yes | na | 0 | Condom | yes | Vaginal ring |
| 11 | M | Urban | C | 39 | Single | 18 | yes | 19 | 1 | No use | yes | Pill |
| 12 | M | Urban | C | 42 | Union | 8 | yes | 19 | 5 | Implant and condom | yes | Injection |
| 13 | M | Urban | C | 40 | Union | 10 | yes | 15 | 4 | Condom (sometimes) | yes | Injection |
| 14 | M | Urban | C | 34 | Divorced | 12 | yes | 24 | 2 | IUD | yes | Pill |
| 15 | M | Rural | HC | 33 | Divorced | 10 | no | 21 | 3 | Condom | yes | Injection and pill |
| 16 | M | Rural | HC | 21 | Union | 6 | no | 15 | 2 | Injection | yes | Injection and pill |
| 17 | M | Rural | HC | 43 | Married | 5 | no | 15 | 6 | Condom | yes | Injection and pill |
| 18 | M | Rural | HC | 26 | Union | 7 | no | 17 | 1 | No use | yes | Condom |
| 19 | M | Rural | HC | 31 | Union | 0 | yes | 16 | 3 | Implant | yes | Pill and condom |
| 20 | M | Rural | HC | 18 | Single | 10 | no | 17 | 1 | Injection and condom | yes | Condom |
| 21 | M | Rural | HC | 23 | Married | 11 | no | 15 | 5 | Pill | yes | Pill |
| 22 | M | Urban | HC | 20 | Union | 10 | yes | 18 | 1 | Injection | yes | Injection and condom |
| 23 | M | Urban | HC | 44 | Married | 8 | no | 16 | 4 | Implant | yes | Pill |
| 24 | M | Urban | HC | 32 | Union | 12 | yes | 25 | 3 | Pill | yes | Pill |
| 25 | M | Urban | HC | 32 | Divorced | 0 | no | 15 | 4 | Pill | yes | Injection and condom |
| 26 | M | Urban | HC | 33 | Married | 12 | yes | 18 | 4 | IUD | yes | Implant and pill |
| 27 | M | Urban | HC | 30 | Union | 10 | yes | 17 | 3 | Pill | yes | Injection |
| 28 | M | Urban | HC | 22 | Single | 9 | no | 18 | 1 | Injection | yes | Implant |
| 29 | M | Urban | C | 42 | Divorced | 12 | yes | 17 | 4 | Condom | no | na |
| 30 | M | Urban | C | 39 | Married | 6 | yes | 17 | 4 | Injection | yes | Pill |
| 31 | M | Urban | C | 47 | Married | 5 | no | 18 | 5 | No use | yes | Injection |
| 32 | M | Urban | C | 37 | Single | 7 | no | 21 | 5 | Injection and condom | yes | IUD |
| 33 | M | Urban | C | 48 | Widow | 7 | no | 20 | 6 | No use | yes | Injection + condoms |
| 34 | M | Urban | C | 20 | Single | 10 | no | 16 | 1 | No use | yes | Injection + condoms |
| 35 | M | Rural | C | 39 | Union | 0 | no | 17 | 7 | No use | yes | Injection |
| 36 | M | Rural | C | 33 | Divorced | 4 | no | 19 | 7 | No use | yes | Pill and implant |
| 37 | M | Rural | C | 25 | Union | 6 | no | 16 | 3 | Condom | yes | Injection and Pill |
| 38 | M | Rural | C | 31 | Union | 6 | no | 16 | 3 | Sterilization | yes | Injection |
| 39 | M | Rural | C | 36 | Union | 8 | no | 14 | 5 | Injection | yes | Pill and implant |
| 40 | N | Urban | HC | 30 | Union | 4 | no | 16 | 6 | Injection | no |  |
| 41 | N | Urban | HC | 36 | Union | 10 | no | 18 | 5 | Injection | yes | Injection |
| 42 | N | Urban | HC | 19 | Union | 11 | no | 19 | 1 | Injection | no |  |
| 43 | N | Urban | HC | 21 | Union | 12 | no | 18 | 1 | Injection | yes | Condom |
| 44 | N | Urban | HC | 23 | Divorced | 12 | yes | 20 | 3 | Injection | no |  |
| 45 | N | Urban | HC | 20 | Single | 12 | yes | na | 0 | Implant | no |  |
| 46 | N | Urban | HC | 40 | Union | 12 | yes | 19 | 6 | Injection | yes | Condom |
| 47 | N | Urban | HC | 18 | Union | 7 | no | 18 | 1 | No use | yes | Condom |
| 48 | N | Urban | HC | 25 | Union | 10 | no | 20 | 3 | Injection | Yes | Implant |
| 49 | N | Urban | HC | 21 | Union | 12 | no | 17 | 2 | Pill | yes | Injection |
| 50 | N | Urban | C | 32 | Union | 10 | no | 17 | 4 | Injection | yes | Condom |
| 51 | N | Urban | C | 46 | Widow | 10 | no | * | 15 | No use | Yes | Injection and pill |
| 52 | N | Urban | C | 47 | Union | 10 | no | 20 | 8 | No use | no |  |
| 53 | N | Urban | C | 35 | Union | 9 | no | 19 | 5 | Injection | yes | Injection |
| 54 | N | Rural | HC | 21 | Single | 9 | no | * | 3 | No use | no |  |
| 55 | N | Rural | HC | 23 | Union | 8 | no | 20 | 2 | No use | yes | Injection |
| 56 | N | Rural | HC | 36 | Union | 0 | no | 18 | 7 | Injection | no |  |
| 57 | N | Rural | HC | 27 | Union | 8 | no | 21 | 5 | Pill | yes | Pill |
| 58 | N | Rural | HC | 18 | Union | 8 | no | 17 | 1 | No use | no |  |
| 59 | N | Rural | HC | 22 | Union | 5 | no | 20 | 2 | Injection | yes | Injection |
| 60 | N | Rural | HC | 19 | Union | 4 | no | 18 | 1 | No use | no |  |
| 61 | N | Rural | HC | 22 | Union | 4 | no | 18 | 4 | No use | no |  |
| 62 | N | Rural | HC | 20 | Single | 0 | no | 19 | 1 | Injection | no |  |
| 63 | N | Rural | C | 44 | Union | 8 | no | 19 | 9 | Injection | yes | Injection |
| 64 | N | Rural | C | 30 | Union | 4 | no | 18 | 12 | No use | yes | Injection, Pill and Condom |

# - interview number; Region: M – Maputo, N-Nampula; Site: HC – Health center, C- Community; *missing
